# Targeting BMP-1 enhances anti-tumoral effects of doxorubicin in metastatic mammary cancer: common and distinct features of TGF-β inhibition

**DOI:** 10.1007/s10549-024-07592-4

**Published:** 2025-01-10

**Authors:** Nuray Erin, Esra Tavşan, Seren Haksever, Azmi Yerlikaya, Chiara Riganti

**Affiliations:** 1 Department of Medical Pharmacology, Faculty of Medicine, Akdeniz University, Antalya, Turkey; 2 Department of Medical Biology, Faculty of Medicine, Kutahya Health Sciences University, Kutahya, Turkey; 3 Department of Oncology, University of Torino, Via Nizza 44, 10126 Turin, Italy; 4 Molecular Biotechnology Center “Guido Tarone”, Via Nizza 44, 10126 Turin, Italy; 5 Interdepartmental Center “G.Scansetti” for the Study of Asbestos and Other Toxic Particulates, University of Torino, 10126 Turin, Italy

**Keywords:** Mammary cancer, Metastasis, TGF-b, Doxorubicin, BMP-1, Drug resistance

## Abstract

**Purpose:**

Mammary carcinoma is comprised heterogeneous groups of cells with different metastatic potential. 4T1 mammary carcinoma cells metastasized to heart (4THM), liver (4TLM) and brain (4TBM) and demonstrate cancer-stem cell phenotype. Using these cancer cells we found thatTGF-β is the top upstream regulator of metastatic process. In addition, secretion of bone morphogenetic protein 1 (BMP-1), which is crucial for the proteolytic release of TGF-β, was markedly high in metastatic mammary cancer cells compared to non-metastatic cells. Although TGF-β inhibitors are in clinical trials, systemic inhibition of TGF-β may produce heavy side effects. We here hypothesize that inhibition of BMP-1 proteolytic activity inhibits TGF-β activity and induces anti-tumoral effects.

**Method and Results:**

Effects of specific BMP-1 inhibitor on liver and brain metastatic murine mammary cancer cells (4TLM and 4TBM), as well as on human mammary cancer MDA-MB-231 and MCF-7 cells, were examined and compared with the results of TGF-β inhibition. Inhibition of BMP-1 activity markedly suppressed proliferation of cancer cells and enhanced anti-tumoral effects of doxorubicin. Inhibition of BMP-1 activity but not of TGF-β activity decreased colony and spheroid formation. Differential effects of BMP-1 and TGF-β inhibitors on TGF-β secretion was also observed.

**Conclusions:**

These results demonstrated for the first time that the inhibition of BMP-1 activity has therapeutic potential for treatment of metastatic mammary cancer and enhances the anti-tumoral effects of doxorubicin.

**Supplementary Information:**

The online version contains supplementary material available at 10.1007/s10549-024-07592-4.

## Introduction

The mortality of mammary cancer, an important cause of cancer-related deaths worldwide [1], is mainly due to metastases of the disease. Mammary carcinoma includes heterogeneous groups of cells with different metastatic potential, which may underlye multi-drug resistance. Small percentage of mammary cancer cells can produce visceral metastasis [2], mainly to the lymph nodes, lung, liver, bone, and brain [3]. Efficacy of major therapeutic approaches is greatly reduced once the cancer cells acquire metastatic properties [4]; hence, distinctive features of metastatic cells should be explored for novel treatment strategies. We previously established differential features of 4T1 mammary carcinoma cells metastasized to heart (termed 4THM), liver (termed 4TLM) and brain (termed 4TBM). These metastatic subsets are more aggressive than 4T1 cells and demonstrate cancer-stem cell phenotype inducing extensive visceral metastasis [5–10]. Among these metastatic cells, cells metastasized to liver (4TLM) induce significantly more metastases *in vivo*, followed by 4TBM and 4THM cells [5, 10].

4T1 is commonly used as a model for triple-negative mammary cancer (TNBC) that carries a high risk for early metastasis and has a poorer prognosis than other mammary cancer subtypes [11]. Since new therapies are urgently needed for metastatic TNBC, metastatic subtypes of 4T1 cells can be a model to explore new therapeutic targets [12]. In accordance, using 4TLM, 4TBM and non-metastatic/hardly metastatic 67NR cells, we previously documented that autocrine factors secreted from metastatic cells may limit anti-tumoral efficiency of chemokine antagonists [13, 14]. Using label-free LC–MS/MS proteomic technique, we also found that eighty-five proteins of over 500 proteins secreted were differentially altered in metastatic cells such that proteins differentially secreted from metastatic cells are involved primarily in carcinogenesis, and TGF-β being the top upstream regulator of metastatic process [6]. Recent findings also highlight TGF-β inhibition as a potent method to target mesenchymal (CD44+/CD24−) and epithelial (ALDHhigh) cancer stem cell (CSC) populations [15].

TGF-β is found mostly in complex with other extracellular matrix (ECM) proteins [16–18]. Our metastatic cells have higher levels of free form of TGF-β, the highest being in 4TLM followed by 4TBM cells. 4TLM cells are highly aggressive, and show an epithelial–mesenchymal transition (EMT) phenotype, with increased cell motility and metastatic potential. This is in accordance with well-established role of TGF-β in EMT [19], which increases metastases of advanced carcinomas [20–23]. The increased free form of TGF-β in metastatic cell lines might be due to extensive secretion of bone morphogenetic protein 1 (BMP-1) from metastatic subsets [6]. In accordance, BMP-1 secretion of non-metastatic 67NR mammary cancer cells was much lower [6].

BMP-1 belongs to the BMP-1/tolloid-like proteinases family and is recognized as key regulator of several developmental and physiological processes [24]. BMP-1 is also one of the ECM proteins associated with high-grade tumors and/or poor prognosis in several types of cancers [25–27]. BMP-1 induces proteolytic maturation of the precursors of more than 30 substrates [28]. Through BMP-1 proteolytic activity, the released TGF-β is complexed with extracellular proteins such as latent TGF-β binding protein–1 (LTBP-1) [29, 30], and increases its activity. TFG-β1 is not only involved in metastatic process but also in drug resistance by inducing EMT [31, 32]. In accordance, it was shown that chemotherapeutic drug doxorubicin activates TGF-β signaling and induces EMT, promoting invasion and generation of cells with stem cell phenotype in murine 4T1 mammary cancer cells [32]. These changes may cause resistance to doxorubicin and increased aggressiveness of the tumor cells following chemotherapy. Hence, we here hypothesize that a marked secretion of BMP-1 by metastatic cells enhances tumor growth and alters drug response. To test this hypothesis we examined the effects of specific BMP-1 inhibitor alone and in combination with doxorubicin on liver and brain metastatic murine mammary cancer cells (4TLM and 4TBM, respectively). These subsets were chosen because each one has distinct features and responds to treatments differently [7, 10]. 4TLM also has intrinsic EMT phenotype suggesting higher influences of TGF-β pathway [5]. In addition, the role of BMP-1 inhibition on MDA-MB-231 metastatic human TNBC cells were determined, while MCF-7 cells were used to model non-metastatic hormone receptor positive human mammary carcinoma.

## Materials and methods

### Cell lines

4 T1 mammary cancer cells were previously derived from a spontaneously formed mammary tumor in a BALB/c female mouse [14]. 4THM cell line was derived from heart metastasis of 4T1 cells. 4TLM cell line was derived from liver metastases and 4TBM cell line was derived from brain metastasis of 4THM cells by Erin et al., [5]. 4TLM and 4TBM cells were grown in DMEM-F12 supplemented with 5% FBS (fetal bovine serum), 2 mM L-glutamine, 1 mM sodium pyruvate, 0.02 mM non-essential amino acids and gentamicin (80 μg/mL).

### Cell proliferation assay

Cells were plated in 96-well plates as 500–750 cells/well, and treated for 72 h with varying concentrations of the inhibitors for acute experiments. Cells were plated as 100–150 cells/well in 96-well plates for multiple treatments without passaging. Specifically, cells were treated with the compounds three times every 72 h. UK383367 (UK) was used as a specific BMP-1 inhibitor, because the IC_50_ of UK is 44 nM for inhibition of BMP-1 activity while it is much higher for inhibition of other metalloproteases [33]. SB-431542 (SB) was chosen to inhibit TGF-β receptor activity [34, 35]. Both inhibitors were obtained from Tocris Bioscience. Doxorubicin is an antineoplastic drug broadly used for the treatment of mammary cancer [8, 36]. Possible changes in sensitivity of chemotherapy was evaluated using PEGylated liposomal doxorubicin (Caelyx). After treatment, the number of viable cells was determined using a WST-1 (Roche Diagnostics GmbH (Mannheim, Germany) cell proliferation assay. Experiments were performed in triplicates under similar experimental conditions.

### Colony formation assay

Cells were plated in a 6-well plated at 200 cells/well density for 4TBM and 4TLM cells. Cells were plated as 500 cells/well for MCF-7 and as 400 cells/well for MDA-MB-231 cells in 24-well plates. Cells were treated with various concentrations of UK (0.1 and 1 μM) and SB (1 and 5 μM) 24 h after plating every 72 h. The colonies were fixed and stained 12–14 days after treatment. Briefly cells were fixed in methanol:acetic acid (3:1) solution and colonies were stained drop by drop with 0.5% crystal violet (in methanol) dye to cover the surface. After 15 min of incubation, the wells were washed with water and left to dry at room temperature. Colonies of approximately 50 cells or more were counted using Azure spot software as well as manually. Colony formation efficiency (CFE) was calculated to determine possible difference in murine metastatic cells and human mammary carcinoma cell lines[37] as it follows: CFE (%) = colonies counted/cell innoculated *100. When the cells reached approximately 90% density, 200 cells/well were seeded in 6-well plates. After 24 h, the cells attached to the bottom of the plate and each treatment was applied in 2 replicates.

Photographs of the colonies were taken. Using the Azure spot colony calculation program on the computer, colonies were marked on the photographs and the number of colonies in each well and the area calculations were made. It was transferred to the Excel program and graphs were created by taking the sum of the areas in the well and the average of the group.

### Formation of spheroid cultures

Spheroid cultures of 4TLM and 4TBM cells were prepared using ultra-low-attachment 96-well plates (Corning, Acton, MA, USA). Cells were seeded as 1000 cells/well and incubated in DMEM-F12 medium including 5% FBS and treated with the inhibitors 16–20 h after seeding. Changes in number of spheroids were determined 5–6 days after seeding using Azure spot software.

### Measurement of TGF-β levels

To determine changes in TGF-β1 levels, cells were grown as colonies and spheroids as described above and TGF-β levels were measured 72 h after treatment. TGF-β levels were measured using the protocol provided by TGF beta-1 Human/Mouse Uncoated ELISA Kit (Invitrogen, Catalog # 88–8350-88).

### Western blot analysis

Because the major changes in cell growth were observed after multiple treatments, we have measured changes in critical intracellular signaling molecules (Akt and Erk) following treatments with the inhibitors 5 to 6 times at 72 h. When required cells were re-plated. Cell lysates were obtained and were separated on 12% polyacrylamide gels under denaturing conditions. The proteins were then transferred to Polyvinylidene Difluoride (PVDF) membrane at 40 V overnight. Dilutions of primary antibodies used were as p-Akt (1:1000, Biorbyt, orb127667 Ser473), p-Erk1/2 (1:1500, Cell Signaling, 9106, Thr202/Tyr204). Proteins were visualized using enhanced chemiluminescence (ECL). GAPDH levels (1:100,000, Meridian Life Science, H86504 M) was used as a housekeeping protein to ensure equal loading and transfer of proteins.

### Statistics

ANOVA with Dunnett’s posttest was used. *p* values < 0.05 were considered biologically significant. When required Student’s t test was used. Statistical analyses were performed using GraphPad InStat 3 software.

## Results

### Acute effects of BMP-1 and TGF-β inhibitor on proliferation of breast carcinoma cells

**Fig. 1:**
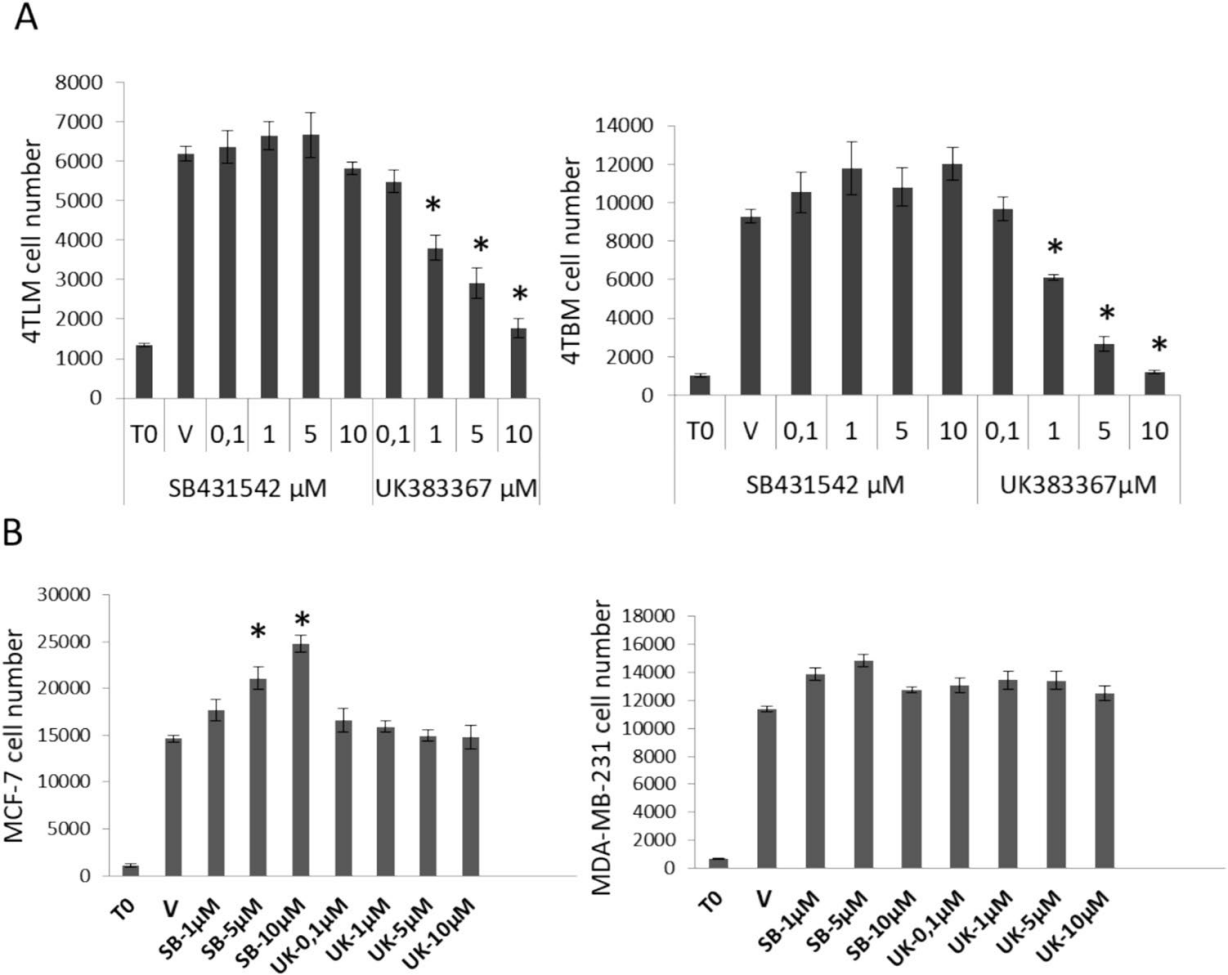
The effects of BMP-1 and TGF-β inhibitors on cell proliferation of murine and human mammary carcinoma cells following 72 h after treatment. Representative results of 3 independent experiments are shown. Cells were treated with the inhibitors once. Panel **A** demonstrates changes in proliferation of metastatic murine mammary carcinoma cells (4TLM and 4TBM). Panel **B** shows the results obtained from human mammary carcinoma cells (MCF-7 and MDA-MB-231). UK383367 (UK) is an inhibitor of BMP-1 and SB431542 (SB) is an inhibitor of TGF-β. *p < 0.05 significantly different from vehicle (V) treated group

We initially determined effects of BMP-1 inhibitor UK and TGF-β inhibitor of SB on proliferation of 4TBM, 4TLM, MDA-MB-231 and MCF-7 cells. As seen in Fig. 1, UK concentration depedently decrease the proliferation of 4TBM and 4TLM cells. On the other hand, SB did not induce major changes in proliferation of 4TBM and 4TLM cells but enhaced proliferation of MCF-7 cells. These results demonstrated that inhibiton of BMP-1 activity decreases cell proliferation independently of TGF-β inhibition. Because BMP-1 inhibition is expected to alter avalibity/function of extracellular proteins, the growth inhibitory effects might be more visible in an extended period of time. Hence we increased the treatment duration.

### Repeated treatment with BMP-1 inhibitor suppresses proliferation of metastatic mammary carcinoma cells and augments anti-proliferative effects of doxorubicin

**Fig. 2:**
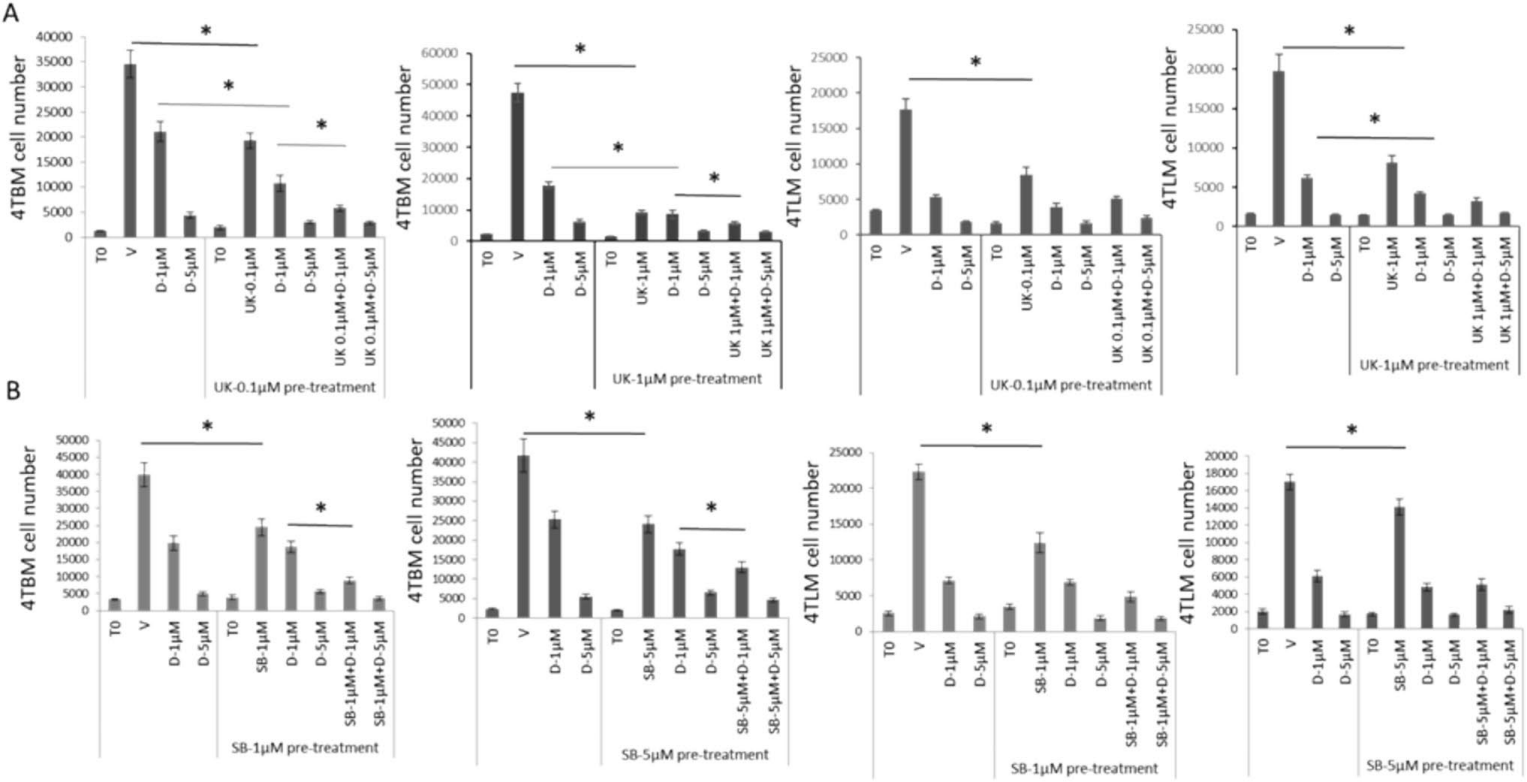
The effects of BMP-1 and TGF-β inhibitors alone or in combination with doxorubicin on proliferation of metastatic mammary carcinoma cells following repeated treatments for a duration of 11–12 days. Cells were treated 3 times without passaging. Experiments were repeated 3–4 times. Panel **A** demonstrates the effects of UK383367 (UK), inhibitor of BMP-1 on 4TBM and 4TLM cells. *p < 0.05 demonstrates anti-proliferative effects of UK alone and UK-dependent increases in anti-proliferative effects of doxorubicin. Panel **B** demonstrates the effects of SB431542 (SB), inhibitor of TGF-β on 4TBM and 4TLM cells. T0 indicates the cell number just before the treatment.*p < 0.05 demonstrates anti-proliferative effects SB alone as well as SB-dependent significant increases in anti-proliferative effects of doxorubicin

Cells were seeded at a lower density (100–150 cells/well) and the first treatment was applied 48 h after seeding. A group of cells were first treated with UK for 72 h (pretreatment), then treated with doxorubicin alone or in combination with UK. The second and third treatments were applied every 72 h. Changes in cell proliferation was determined 72 h after the last treatment. Two different concentrations of UK (0.1 and 1 µM) were used for pretreatments. As seen in Fig. 2A, lower concentrations of UK were effective when treatment duration was increased four times. Furthermore, pretreatment with UK markedly enhanced anti-proliferative effects of doxorubicin. Both concentrations of UK markedly enhanced the anti-proliferative effects of lower concentration of doxorubicin (1 µM) in 4TBM cells while similar changes were observed in 4TLM cells following pretreatment with higher concentration. UK alone at these concentrations was as effective as doxorubicin in suppressing cell proliferation especially in 4TBM cells. Similarly, combination of doxorubicin (1 µM) with UK also suppressed cell proliferation more effectively compared to single treatment (Fig. 2A).

### TGF-β inhibitor suppresses proliferation of metastatic mammary carcinoma cells but does not augment anti-proliferative effects of doxorubicin

We next examined the effects of multiple treatments (3) with SB on cell proliferation. SB significantly suppressed cell proliferation similarly to UK. Combination of doxorubicin with SB suppressed cell proliferation more effectively compared to single treatment in 4TBM cells. Differently, pretreatment with SB (1 and 5 µM) did not enhance the anti-proliferative effects of doxorubicin (Fig. 2B). Although BMP-1 inhibitors prevent activation of stromal TGF-β, the distinct effects of UK observed demonstrated that anti-proliferative effect of UK is not solely related to inhibition TGF-β activity.

### Inhibition of BMP-1 activity but not TGF-β activity decreases colony and spheroid formation of metastatic mammary carcinoma cells

**Fig. 3:**
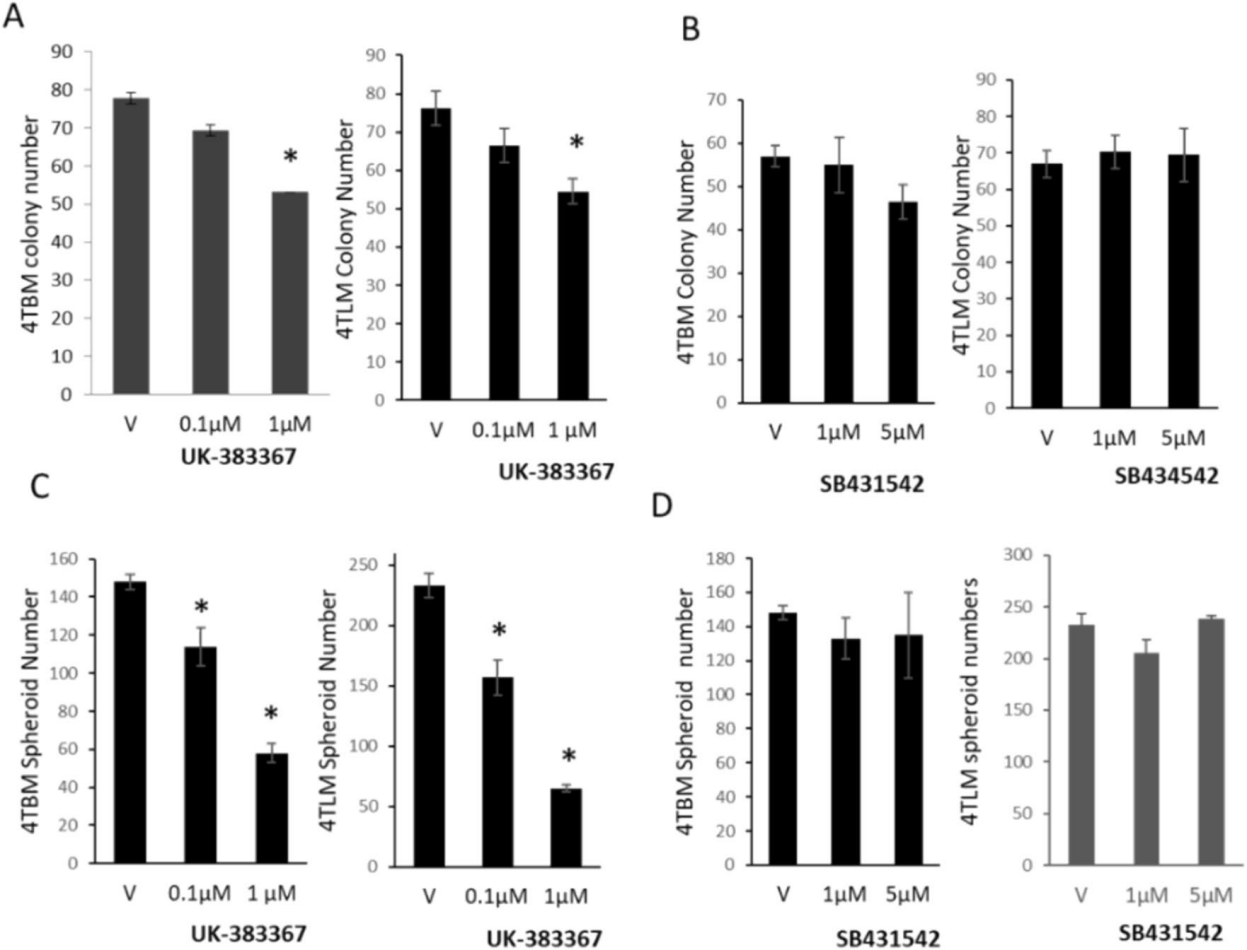
The effects of BMP-1 and TGF-β inhibitors on colony and spheroid formation of metastatic mammary carcinoma cells. The effects of UK383367 (UK), inhibitor of BMP-1 (Panel **A**) and SB431542 (SB), inhibitor of TGF-β (Panel **B**) on colony formation are seen (cells were treated 2–3 times every 72 h.). Panel **C**, and **D** demonstrates the effects of UK and SB on spheroid formation, respectively (cells were treated once). *p < 0.05 significantly different than vehicle (V)-treated group. Experiments were repeated 3–4 times

We next determined possible differences of BMP-1 and TGF-β inhibition on colony and spheroid formation. Additional growth factor such as B27, FGF and insulin are required for spheroid formation for most cancer cells [38]. 4TLM and 4TBM cells, however, are highly aggressive and even in the absence of additional growth factors, effectively form spheroids (Supp. Fig.) in ultra-low-attachment plates. As seen in Fig. 3A, B, UK concentration-dependently decreased both colony and spheroid formation. Interestingly, UK was more effective in decreasing spheroid formation. SB, on the other hand, did not alter colony as well as spheroid formation (Fig. 3C, D) (representative pictures of colony formation is shown in Supp. Fig.).

### Differential effects of BMP-1 and TGF-β inhibitors on TGF-β secretion as well as activation of AKT and Erk in brain and liver metastatic cells

**Fig. 4:**
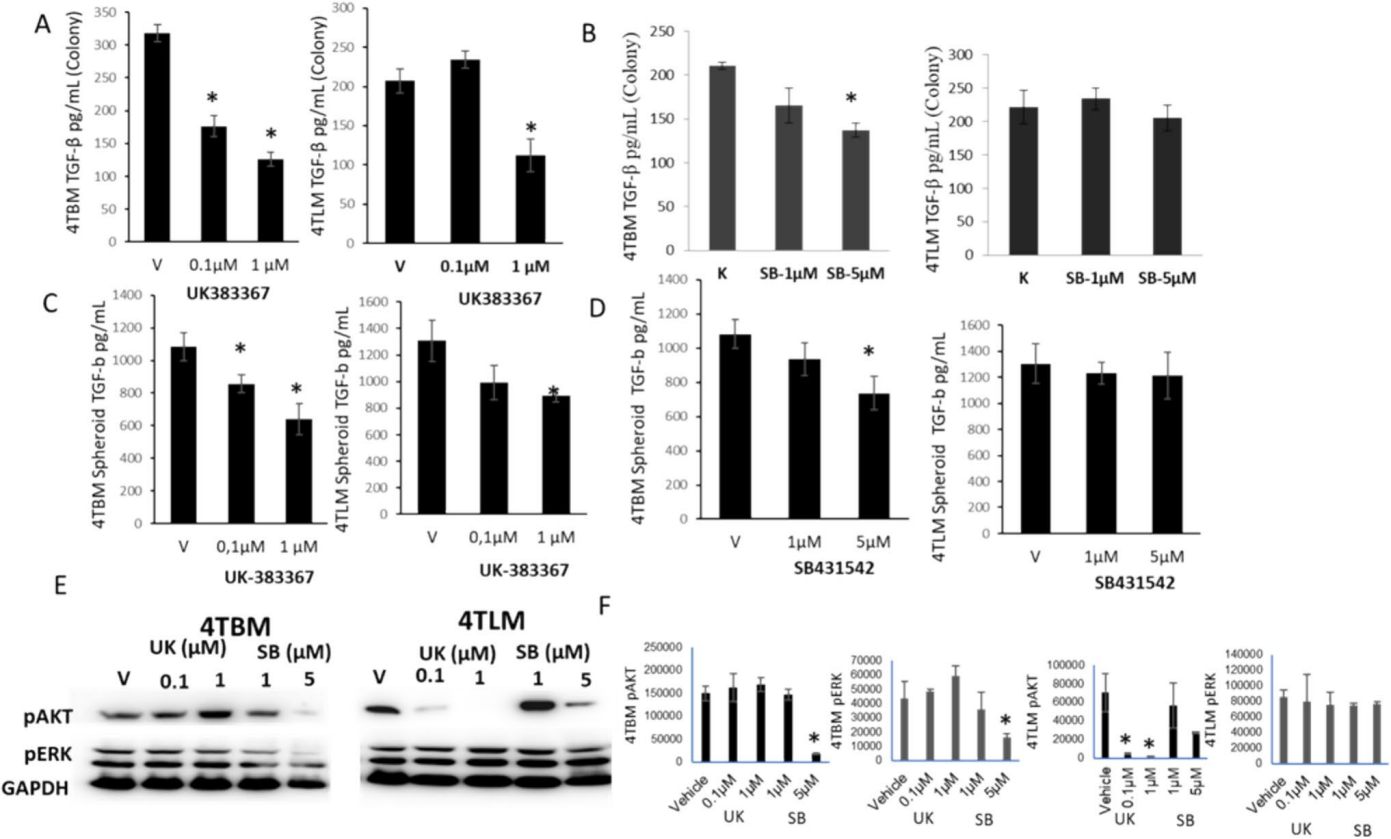
The effects of BMP-1 and TGF-β inhibitors on TGF-β secretion, AKT and Erk activation of metastatic mammary carcinoma cells. The effects of UK383367 (UK), inhibitor of BMP-1 on TGF-β secretion from colonies of 4TBM and 4TLM cells are seen on Panel **A** (cells were treated 2–3 times every 72 h.). Panel **B** demonstrates the effects of SB-431542 (SB), inhibitor of TGF-β on TGF-β secretion of 4TBM and 4TLM cells. Panel **C** and **D** shows the effects of BMP-1 and TGF-β inhibitors on TGF-β release from spheroids formed by metastatic mammary carcinoma cells (4TLM and 4TBM) (cells were treated once). Panel **E** and **F** demonstrates immunoblots of pAkt, pErk and Gapdh as well as relative density of 3 independent experiments in 4TBM and 4TLM cells following treatment with UK and SB. UK (UK383367) is an inhibitor of BMP-1 and SB (SB431542) is an inhibitor of TGF-β. Experiments were repeated 3 times. *p < 0.05 significantly different from vehicle (V) treated group

BMP-1 activates TGF-β by releasing it from extracellular complex and may alter TGF-β levels. Hence we evaluated changes in TGF-β secretion from colonies as well as spheroids. UK decreased TGF-β secretion in both 4TBM and 4TLM cells; the effect, however, was more prominent in 4TBM cells. Inhibition of TGF-β activity by SB decreased TGF-β secretion only in 4TBM cells in colonies (Fig. 4A, B). Spheroids secreted markedly higher levels of TGF-β and treatment with UK and SB-induced similar changes as seen in Fig. 4C, D.

Changes in AKT and Erk activation were determined after repeated treatment with the inhibitors. Surprisingly BMP-1 inhibitor decreased AKT phosphorylation in a concentration-dependent manner only in 4TLM cells while TGF-β inhibitor suppressed AKT phosphorylation in both cell lines. Erk phosphorylationwas not markedly affected by the inhibitors (Fig. 4E, F).

### Inhibition of BMP-1 activity markedly decreases cell proliferation in human triple negative mammary carcinoma cells (MDA-MB-231) and enhances anti-proliferative effects of doxorubicin

**Fig. 5:**
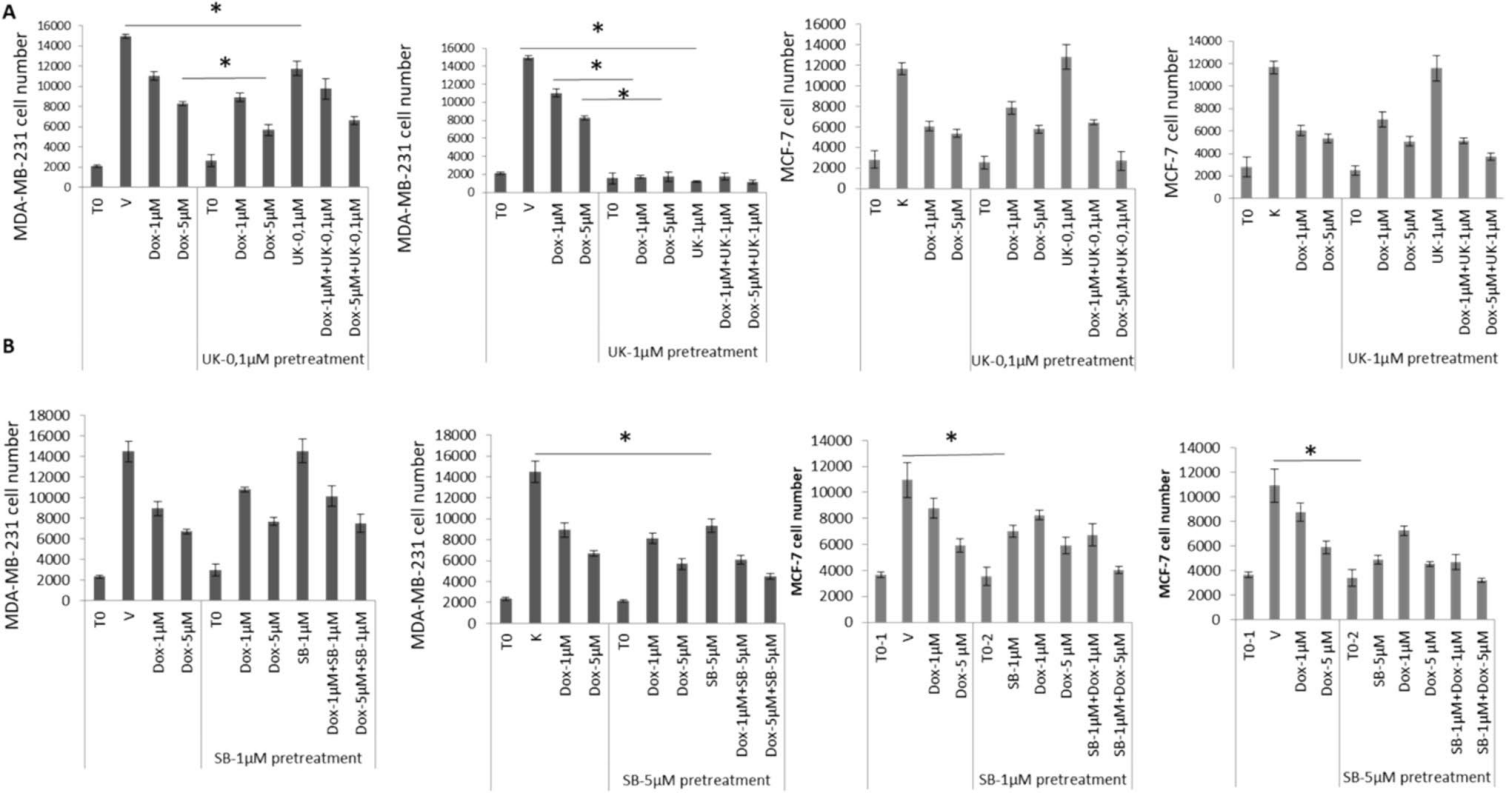
The effects of BMP-1 and TGF-β inhibitors alone or in combination with doxorubicin on proliferation of MDA-MB-231 and MCF-7 cells following repeated treatments for a duration of 11–12 days. Panel **A** demonstrates the effects of UK383367 (UK), inhibitor of BMP-1. *p < 0.05 demonstrates anti-proliferative effects of UK alone and UK-dependent increases in the anti-proliferative effects of doxorubicin. Panel **B** demonstrates the effects of SB-431542 (SB), inhibitor of TGF-β. T0 denotes the cell number just before the treatment. Cells were treated 3 times without passaging. Experiments repeated 3–4 times *p < 0.05 demonstrates anti-proliferative effects SB alone

We here selected MDA-MB-231 cell line to model human triple negative mammary carcinoma which is also commonly used to mimic the metastatic disease. MCF-7 (Estrogen/progesterone receptor positive ER/PR+, luminal A molelular subtype) cell line has low metastatic potential [39], hence it was used here to represent poorly aggressive and noninvasive cell line. As seen in Fig. 1, the anti-proliferative effects of UK were not remarkable within 72 h of treatment in both cell lines. A major suppressive effect, however, was observed when the duration of treatment increased in MDA-MB-231 cells. Specifically UK at 1 µM concentration completely suppressed cell proliferation and markedly enhanced anti-proliferative effects of doxorubicin. At1 µM, doxorubicin completely inhibited cell proliferation of cells pretreated with UK 1 µM while its suppressive effects was approximately 25% in untreated MDA-MB-231 cells. Co-administration of doxorubicin with UK did not further inhibit the cell proliferation. Similar changes were observed in MDA-MB-231 cells treated with 0.1 µM UK but the effect was less prominent (Fig. 5A). We did not observe anti-proliferative effects of UK in MCF-7 cells after acute as well as extended periods of treatments (Fig. 5A and Fig. 1) suggesting that BMP-1 plays a cancer promoting role in aggressive cells that have metastatic potential. Doxorubicin effectively suppressed cell proliferation, but pretreatment with UK did not enhance anti-proliferative effects of Doxorubicin in MCF-7 cells (Fig. 5A).

### Inhibition of TGF-β activity markedly decreases cell proliferation in human mammary carcinoma cell, an effect more prominent in non-metastatic ER + and PR + MCF-7 cells

Anti-proliferative effects of SB were not remarkable within 72 h of treatment in both MDA-MB-231 and MCF-7 cell lines. On the contrary, inhibition of TGF-β activity increased cell proliferation in MCF-7 cells within 72 h as described above (Fig. 1). This may reflect anti-proliferative effects of TGF-β in the early stages of carcinogenesis as MCF-7 cells model. Treatment with SB for a longer period of time, on the other hand, markedly suppressed cell proliferation in MCF-7 cells at low concentration. Higher concentration was required to observe this effect in MDA-MB-231 cells. Pretreatment with SB, however, did not enhance the anti-proliferative effects of doxorubicin (Fig. 5B).

### Inhibition of BMP-1 and TGF-β activity decreases colony formation and TGF-β secretion in MDA-MB-231 and MCF-7 cells

**Fig. 6:**
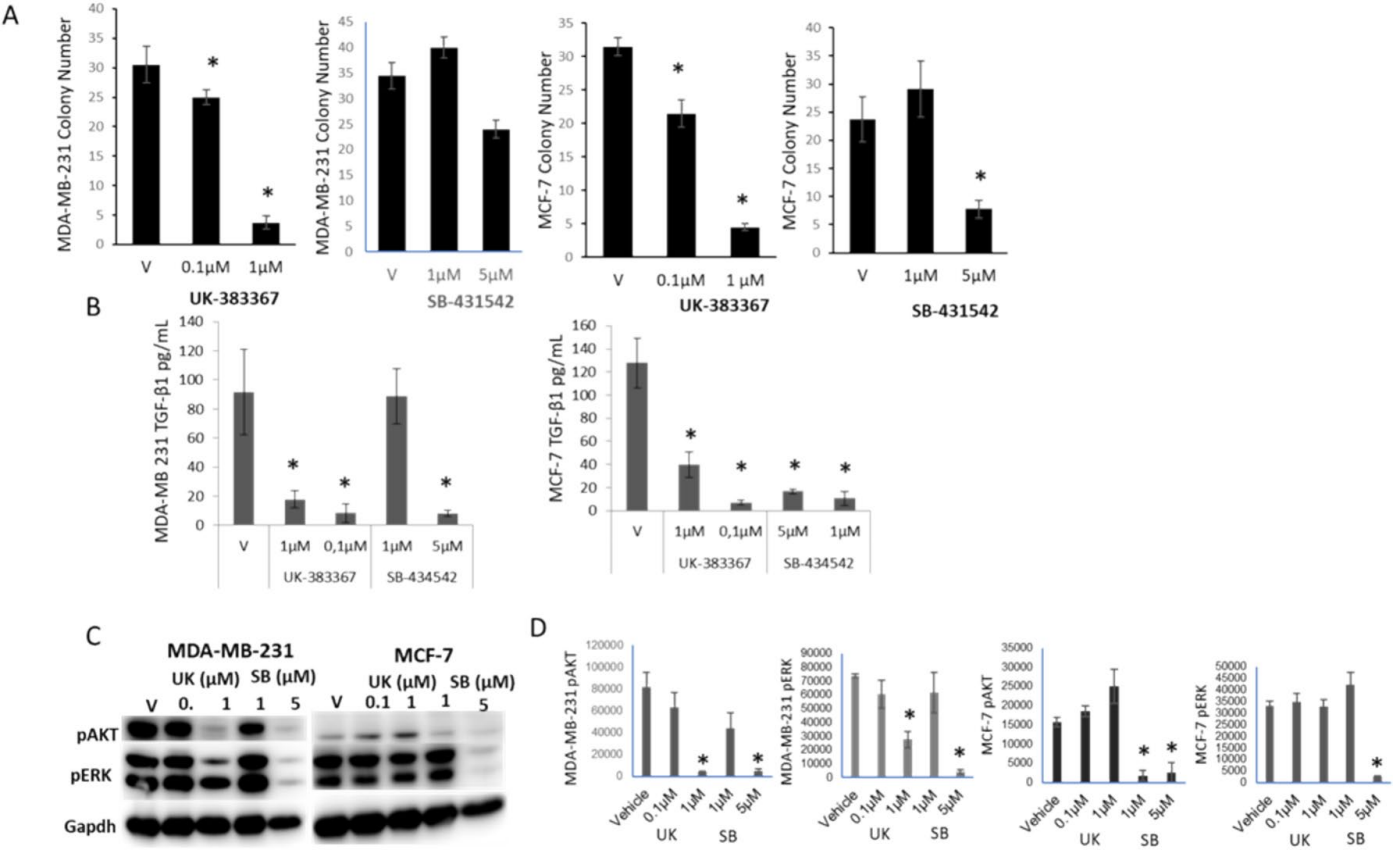
The effects of BMP-1 and TGF-β inhibitors on colony formation, TGFb secretion as well as AKT and Erk activation of MDA-MB-231 and MCF-7 cells. The effects of UK383367 (UK), inhibitor of BMP-1 and SB-431542 (SB), inhibitor of TGF-β, on colony formation (Panel **A**) and TGF-β secretion (Panel **B**) are seen. Experiments repeated 3–4 times. Panel **C** demonstrates immunoblots of pAKT, pErk and Gapdh following treatment with UK and SB and Panel **D** shows densitometric analysis of western blots from 3 independent experiments. *p < 0.05 compared to vehicle (V) group

UK dose-dependently suppressed colony formation as well as TGF-β secretion in both cell lines, similar to 4TLM and 4TBM cells (Fig. 6). Differently, SB also suppressed colony formation in MDA-MB-231 and MCF7 cells. SB also markedly lowered TGF-β secretion in these cells while it was much less effective in 4TBM and 4TLM cells. These differences might be due to the less aggressive features of MDA-MB-231 and MCF7 cells since their CFE was markedly lower. Specifically, CFE was between 30 and 38% for 4TBM and 4TLM cells while it was 5 to 7% for MCF-7 and MDA-M-231 cells. In accordance, MDA-MB-231 and MCF-7 cells did not form spheroids in low-attachment plates in DMEM-F12 medium containing 10% FBS. Hence, 4TLM and 4TBM cells demonstrated markedly more aggressive behavior.

Differences in intracellular signaling response were also present: both UK and SB markedly suppressed AKT and Erk phosphorylation in MDA-MB-231 cells. The active form of AKT was markedly lower in MCF-7 cells in accordance with their less aggressive nature, hence we did not observe major changes. Erk phosphorylation was also markedly suppressed, following treatment with SB in MCF-7 cells (Fig. 6).

## Discussion

BMP-1 is an ECM protein associated with high-grade tumors and/or poor prognosis in several types of cancers [25–27]. Our results suggest that association of BMP-1 with high grade tumors and poor prognosis might be due to increased survival of cancer cells and resistance to chemotherapy. Specifically, inhibition of BMP-1 activity markedly enhanced anti-proleferative effects of doxorubicin, a commonly used chemotherapeutic drug, in both highly metastatic murine mammary and human mammary cancer cells. BMP-1 inhibitor alone was as effective as doxorubicin in suppressing cell proliferation and inhibited both colony and spheroid formation of 4TLM and 4TBM cells. Similar changes were also observed with MDA-MB-231 and MCF7 cells.

BMP-1 substrates consist of ECM precursor proteins such procollagens I to III, which can form fibers and three-dimensional networks upon proteolytic maturation [28]. Latent forms of growth factors and cytokines such as growth differentiation factor–11 (GDF-11), TGF-β and as well as insulin-like growth factor–binding protein 3 (IGFBP3), which can bind and sequester IGF-I and IGF-II, can be cleaved by BMP-1 to release the active growth factors [40, 41]. BMP-1 cleaves matricellular glycoprotein thrombospondin-1 (TSP-1) between the VWFC/procollagen-like domain and the type 1 repeats that mediate key functions of TSP-1. Furthermore, BMP-1-dependent proteolysis potentiates the TSP-1-mediated activation of latent TGF-β [24] and represents another mechanism by which BMP-1 enhances TGF-β signaling [29, 30]. BMP-1 also responsible for processing lysyl oxidase (LOX) into the active form [42].

Although BMP-1-mediated activation of TGF-β signaling might play an important role in tumor-promoting effects of BMP-1 and consequently, BMP-1 inhibitors should have anti-tumoral effects, our results demonstrate that mechanisms other than TGF-β activation are also involved. Among BMP-1 substrates several of them are associated with the progression and metastasis of cancer. For example, type I and type III collagen as well as TSP-1 are involved in migration of cancer cells [43, 44]. TSP-1 through its integrin receptor α3β1 induces migration of cancer cells and enhances metastasis of mammary cancer [45]. TSP-1 expression is also associated with poor prognosis in various cancers including mammary cancer [46]. LOX, activated by BMP-1 [25], causes stiffening of the matrix and enhances the invasive and metastatic properties of cancer cells [47]. LOX is also associated with mammary cancer bone metastasis through leading to osteolytic lesion formation [48, 49].

We here observed that inhibition of TGF-β and BMP-1 activity induces only partially similar effects in metastatic murine mammary carcinoma cells as well as MDA-MB-231 and MCF-7 human mammary cancer cells. Strikingly, BMP-1 inhibitor had markedly stronger effect on highly metastatic 4TLM and 4TBM cells while TGF-β inhibition was more effective in non-metastatic ER+ and PR+ MCF-7 cells. Importantly inhibition of BMP-1 activity enhanced anti-tumoral effects of doxorubicin in both murine and human metastatic mammary cancer cells, while we did not observe similar changes following TGF-β inhibition. These results suggest that BMP-1 activity is more detrimental in highly aggressive metastatic cancer cells than non-metastatic tumor cells, which may respond BMP-1 inhibition differently.

Few studies documented direct tumor-promoting features of BMP-1. For example, knockdown of BMP-1 suppresses malignancy in renal cell carcinoma. In accordance, high expression of BMP-1 was associated with EMT, angiogenesis, hypoxia pathway, KRAS signaling and shorter overall survival in renal cell carcinoma [27]. Similarly suppression of BMP-1 expression reduces the activity of TGF-β, downregulates MMP2/MMP9 expression and decreases the invasion of lung cancer cells [50]. Our results further support these finding and demonstrate tumor-promoting role of BMP-1 in highly aggressive metastatic mammary carcinoma.

Although doxorubicin is used commonly for mammary cancer treatment, approximately one-third of patients develop drug resistance with a poor prognosis [51]. Doxorubicin induces EMT as shown in 4T1 mammary cancer cells [32]. As explained in method Sect. 4 TBM and 4TLM cells were metastatic subset of 4T1 cells. It was also shown that pre-treatment of 4T1 cells with TGF-β induces doxorubicin resistance [32] and combination of doxorubicin with TGF-β inhibitor enhances anti-tumoral effects of doxorubicin. TGF-β inhibitor here did not enhance the anti-proliferative effects of doxorubicin: this might be due to relatively limited duration of pretreatment. However, we observed marked increases in anti-proliferative effects of doxorubicin following 72 h pretreatment with BMP-1 inhibitor in 4TLM, 4TBM and MDA-MB-231 cells.

It was shown that TGF-β2 increases BMP-1 secretion [52]: indeed, we found that BMP-1 inhibition decreases TGF-β secretion from both human and murine mammary cancer cells demonstrating a positive feed-back among BMP-1 and TGF-β. Interestingly, TGF-β seems to be enhancing its own secretion from mammary cancer cells because inhibition of TGF-β activity also suppressed TGF-β secretion.

Tumor promoting effects of TGF-β is due to both induction of EMT and impairment of cytotoxic immune response [53–58]. The oncogenic activation of noncanonical TGF-β pathways i.e., phosphoinositide-3-kinase (PI3K), AKT, and mTOR are mutually associated with enhancing the proliferation of mammary cancer cells [59]. MAP Kinase activation is considered to be a major mechanism by which TGF-β induces EMT [60]. Activation of p38 MAPK, and JNK (c-Jun N-terminal kinase) pathways by TGF-β may also mediate tumor-promoting features [61]. Here we determined the changes in AKT and Erk activation following treatment with TGF-β and BMP-1 inhibitors for an extended period of time. In accordance with the previous findings, inhibition of TGF-β activity markedly suppressed AKT phosphorylation in 4TLM, 4TBM and MDA-MB-231 cells. TGF-β inhibition also markedly suppressed Erk activity in both MDA-MB-231 and MCF-7 cells. It, however, did not alter Erk phosphorylation in 4TBM and 4TLM cells demonstrating the presence of redundant or confronted factors in these highly aggressive metastatic mammary cancer cells. BMP-1 inhibitor also suppressed AKT phosphorylation in MDA-MB-231 and 4TLM cells though its effect on Erk was limited. These results further demonstrated common and distinct features of TGF-β and BMP-1 inhibition pointing the importance of other BMP-1 targeted pathways.

4TLM and 4TBM as well as MDA-MB-231 cells are models of aggressive TNBC. It was shown that elevated expression of TGF-β was associated with TNBC progression and a reduced disease-free survival [62]. TGF-β suppresses anti-tumoral immunity [58, 63] and clinical trials with TGF-β inhibitors are suggestive of their potential role in the treatment of mammary cancer [63]. On the other hand, TGF-β has pleiotropic nature and effects of systemic inhibition may lead to heavy side effects [64]. TGF-β is a multifunctional cytokine of key importance to the maintenance of tissue homeostasis and targeting of TGF-β signaling has been associated with on-target cardiovascular toxic side effects and formation of benign tumors [65]. Hence, it seems crucial to modulate TGF-β activity within tumor microenvironment. As suggested by the results presented here, BMP-1 inhibitors might serve to limit TGF-β activity, which in turn may target other carcinogenic/metastatic pathways in tumors secreting high concentration of BMP-1.

## Supplementary Information

Below is the link to the electronic supplementary material.

Supplementary file1 (PDF 726 KB)

## Data Availability

No datasets were generated or analysed during the current study.

## References

[1] Bray F, Ferlay J, Soerjomataram I, Siegel RL, Torre LA, Jemal A (2018) Global cancer statistics 2018: GLOBOCAN estimates of incidence and mortality worldwide for 36 cancers in 185 countries. CA Cancer J Clin 68(6):394–424 30207593 10.3322/caac.21492

[2] Valastyan S, Weinberg RA (2011) Tumor metastasis: molecular insights and evolving paradigms. Cell 147(2):275–292 22000009 10.1016/j.cell.2011.09.024 PMC3261217

[3] Hess KR, Varadhachary GR, Taylor SH, Wei W, Raber MN, Lenzi R, Abbruzzese JL (2006) Metastatic patterns in adenocarcinoma. Cancer 106(7):1624–1633 16518827 10.1002/cncr.21778

[4] Sakorafas GH, Tsiotou AG (2000) Ductal carcinoma in situ (DCIS) of the breast: evolving perspectives. Cancer Treat Rev 26(2):103–125 10772968 10.1053/ctrv.1999.0149

[5] Erin N, Kale S, Tanriover G, Koksoy S, Duymus O, Korcum AF (2013) Differential characteristics of heart, liver, and brain metastatic subsets of murine breast carcinoma. Breast Cancer Res Treat 139(3):677–689 23760857 10.1007/s10549-013-2584-0

[6] Erin N, Ogan N, Yerlikaya A (2018) Secretomes reveal several novel proteins as well as TGF-beta1 as the top upstream regulator of metastatic process in breast cancer. Breast Cancer Res Treat 170(2):235–250 29557524 10.1007/s10549-018-4752-8

[7] Erin N, Podnos A, Tanriover G, Duymus O, Cote E, Khatri I, Gorczynski RM (2015) Bidirectional effect of CD200 on breast cancer development and metastasis, with ultimate outcome determined by tumor aggressiveness and a cancer-induced inflammatory response. Oncogene 34(29):3860–3870 25263452 10.1038/onc.2014.317

[8] Erin N, Tanriover G, Curry A, Akman M, Duymus O, Gorczynski R (2018) CD200fc enhances anti-tumoral immune response and inhibits visceral metastasis of breast carcinoma. Oncotarget 9(27):19147–19158 29721190 10.18632/oncotarget.24931 PMC5922384

[9] Erin N, Dilmac S, Curry A, Duymus O, Tanriover G, Prodeus A, Gariepy J, Gorczynski RM (2020) CD200 mimetic aptamer PEG-M49 markedly increases the therapeutic effects of pegylated liposomal doxorubicin in a mouse model of metastatic breast carcinoma: an effect independent of CD200 receptor 1. Cancer Immunol Immunother 69(1):103–114 31811336 10.1007/s00262-019-02444-3 PMC11027860

[10] Nizam E, Koksoy S, Erin N (2020) NK1R antagonist decreases inflammation and metastasis of breast carcinoma cells metastasized to liver but not to brain; phenotype-dependent therapeutic and toxic consequences. Cancer Immunol Immunother 69(8):1639–1650 32322911 10.1007/s00262-020-02574-z PMC11027630

[11] Schrors B, Boegel S, Albrecht C, Bukur T, Bukur V, Holtstrater C, Ritzel C, Manninen K, Tadmor AD, Vormehr M et al (2020) Multi-omics characterization of the 4T1 murine mammary gland tumor model. Front Oncol 10:1195 32793490 10.3389/fonc.2020.01195 PMC7390911

[12] Dillekas H, Rogers MS, Straume O (2019) Are 90% of deaths from cancer caused by metastases? Cancer Med 8(12):5574–5576 31397113 10.1002/cam4.2474 PMC6745820

[13] Erin N, Nizam E, Tanriover G, Koksoy S (2015) Autocrine control of MIP-2 secretion from metastatic breast cancer cells is mediated by CXCR2: a mechanism for possible resistance to CXCR2 antagonists. Breast Cancer Res Treat 150(1):57–69 25682075 10.1007/s10549-015-3297-3

[14] Aslakson CJ, Miller FR (1992) Selective events in the metastatic process defined by analysis of the sequential dissemination of subpopulations of a mouse mammary tumor. Cancer Res 52(6):1399–1405 1540948

[15] Sulaiman A, McGarry S, Chilumula SC, Kandunuri R, Vinod V (2021) Clinically translatable approaches of inhibiting TGF-beta to target cancer stem cells in TNBC. Biomedicines. 10.3390/biomedicines9101386 34680503 10.3390/biomedicines9101386 PMC8533357

[16] Du F, Li J, Zhong X, Zhang Z, Zhao Y (2024) Endothelial-to-mesenchymal transition in the tumor microenvironment: roles of transforming growth factor-beta and matrix metalloproteins. Heliyon 10(21):e40118 39568849 10.1016/j.heliyon.2024.e40118 PMC11577214

[17] Giarratana AO, Prendergast CM, Salvatore MM, Capaccione KM (2024) TGF-beta signaling: critical nexus of fibrogenesis and cancer. J Transl Med 22(1):594 38926762 10.1186/s12967-024-05411-4 PMC11201862

[18] Huang H, Tang Q, Li S, Qin Y, Zhu G (2024) TGFBI: A novel therapeutic target for cancer. Int Immunopharmacol 134:112180 38733822 10.1016/j.intimp.2024.112180

[19] Liu GL, Yang HJ, Liu T, Lin YZ (2014) Expression and significance of E-cadherin, N-cadherin, transforming growth factor-beta1 and Twist in prostate cancer. Asian Pac J Trop Med 7(1):76–82 24418088 10.1016/S1995-7645(13)60196-0

[20] Bierie B, Moses HL (2010) Transforming growth factor beta (TGF-beta) and inflammation in cancer. Cytokine Growth Factor Rev 21(1):49–59 20018551 10.1016/j.cytogfr.2009.11.008 PMC2834863

[21] Sheen YY, Kim MJ, Park SA, Park SY, Nam JS (2013) Targeting the transforming growth factor-beta signaling in cancer therapy. Biomol Ther (Seoul) 21(5):323–331 24244818 10.4062/biomolther.2013.072 PMC3825194

[22] Padua D, Massague J (2009) Roles of TGFbeta in metastasis. Cell Res 19(1):89–102 19050696 10.1038/cr.2008.316

[23] Katz LH, Li Y, Chen JS, Munoz NM, Majumdar A, Chen J, Mishra L (2013) Targeting TGF-beta signaling in cancer. Expert Opin Ther Targets 17(7):743–760 23651053 10.1517/14728222.2013.782287 PMC3745214

[24] Anastasi C, Rousselle P, Talantikite M, Tessier A, Cluzel C, Bachmann A, Mariano N, Dussoyer M, Alcaraz LB, Fortin L et al (2020) BMP-1 disrupts cell adhesion and enhances TGF-beta activation through cleavage of the matricellular protein thrombospondin-1. Sci Signal. 10.1126/scisignal.aba3880 32636307 10.1126/scisignal.aba3880

[25] da Silva R, Uno M, Marie SK, Oba-Shinjo SM (2015) LOX expression and functional analysis in astrocytomas and impact of IDH1 mutation. PLoS ONE 10(3):e0119781 25790191 10.1371/journal.pone.0119781 PMC4366168

[26] Hsieh YY, Tung SY, Pan HY, Yen CW, Xu HW, Deng YF, Lin YJ, Hsu WT, Wu CS, Li C (2018) Upregulation of bone morphogenetic protein 1 is associated with poor prognosis of late-stage gastric Cancer patients. BMC Cancer 18(1):508 29720137 10.1186/s12885-018-4383-9 PMC5930761

[27] Xiao W, Wang X, Wang T, Xing J (2020) Overexpression of BMP-1 reflects poor prognosis in clear cell renal cell carcinoma. Cancer Gene Ther 27(5):330–340 31155610 10.1038/s41417-019-0107-9 PMC7237353

[28] Vadon-Le Goff S, Hulmes DJ, Moali C (2015) BMP-1/tolloid-like proteinases synchronize matrix assembly with growth factor activation to promote morphogenesis and tissue remodeling. Matrix Biol 44–46:14–23 25701650 10.1016/j.matbio.2015.02.006

[29] Delolme F, Anastasi C, Alcaraz LB, Mendoza V, Vadon-Le Goff S, Talantikite M, Capomaccio R, Mevaere J, Fortin L, Mazzocut D et al (2015) Proteolytic control of TGF-beta co-receptor activity by BMP-1/tolloid-like proteases revealed by quantitative iTRAQ proteomics. Cell Mol Life Sci 72(5):1009–1027 25260970 10.1007/s00018-014-1733-x PMC11113849

[30] Ge G, Greenspan DS (2006) BMP-1 controls TGFbeta1 activation via cleavage of latent TGFbeta-binding protein. J Cell Biol 175(1):111–120 17015622 10.1083/jcb.200606058 PMC2064503

[31] Erin N, Grahovac J, Brozovic A, Efferth T (2020) Tumor microenvironment and epithelial mesenchymal transition as targets to overcome tumor multidrug resistance. Drug Resist Updat 53:100715 32679188 10.1016/j.drup.2020.100715

[32] Bandyopadhyay A, Wang L, Agyin J, Tang Y, Lin S, Yeh IT, De K, Sun LZ (2010) Doxorubicin in combination with a small TGFbeta inhibitor: a potential novel therapy for metastatic breast cancer in mouse models. PLoS ONE 5(4):e10365 20442777 10.1371/journal.pone.0010365 PMC2860989

[33] Fish PV, Allan GA, Bailey S, Blagg J, Butt R, Collis MG, Greiling D, James K, Kendall J, McElroy A et al (2007) Potent and selective nonpeptidic inhibitors of procollagen C-proteinase. J Med Chem 50(15):3442–3456 17591762 10.1021/jm061010z

[34] Liu IM, Schilling SH, Knouse KA, Choy L, Derynck R, Wang XF (2009) TGFbeta-stimulated Smad1/5 phosphorylation requires the ALK5 L45 loop and mediates the pro-migratory TGFbeta switch. EMBO J 28(2):88–98 19096363 10.1038/emboj.2008.266 PMC2634733

[35] Inman GJ, Nicolas FJ, Callahan JF, Harling JD, Gaster LM, Reith AD, Laping NJ, Hill CS (2002) SB-431542 is a potent and specific inhibitor of transforming growth factor-beta superfamily type I activin receptor-like kinase (ALK) receptors ALK4, ALK5, and ALK7. Mol Pharmacol 62(1):65–74 12065756 10.1124/mol.62.1.65

[36] Tacar O, Sriamornsak P, Dass CR (2013) Doxorubicin: an update on anticancer molecular action, toxicity and novel drug delivery systems. J Pharm Pharmacol 65(2):157–170 23278683 10.1111/j.2042-7158.2012.01567.x

[37] Runden-Pran E, Mariussen E, El Yamani N, Elje E, Longhin EM, Dusinska M (2022) The colony forming efficiency assay for toxicity testing of nanomaterials-modifications for higher-throughput. Front Toxicol 4:983316 36157975 10.3389/ftox.2022.983316 PMC9489936

[38] Manuel Iglesias J, Beloqui I, Garcia-Garcia F, Leis O, Vazquez-Martin A, Eguiara A, Cufi S, Pavon A, Menendez JA, Dopazo J, Martin AG (2013) Mammosphere formation in breast carcinoma cell lines depends upon expression of E-cadherin. PLoS ONE 8(10):e77281 24124614 10.1371/journal.pone.0077281 PMC3790762

[39] Comsa S, Cimpean AM, Raica M (2015) The story of MCF-7 breast cancer cell line: 40 years of experience in research. Anticancer Res 35(6):3147–3154 26026074

[40] Ge G, Hopkins DR, Ho WB, Greenspan DS (2005) GDF11 forms a bone morphogenetic protein 1-activated latent complex that can modulate nerve growth factor-induced differentiation of PC12 cells. Mol Cell Biol 25(14):5846–5858 15988002 10.1128/MCB.25.14.5846-5858.2005 PMC1168807

[41] Kim B, Huang G, Ho WB, Greenspan DS (2011) Bone morphogenetic protein-1 processes insulin-like growth factor-binding protein 3. J Biol Chem 286(33):29014–29025 21697095 10.1074/jbc.M111.252585 PMC3190709

[42] Maruhashi T, Kii I, Saito M, Kudo A (2010) Interaction between periostin and BMP-1 promotes proteolytic activation of lysyl oxidase. J Biol Chem 285(17):13294–13303 20181949 10.1074/jbc.M109.088864 PMC2857065

[43] Graf F, Horn P, Ho AD, Boutros M, Maercker C (2021) The extracellular matrix proteins type I collagen, type III collagen, fibronectin, and laminin 421 stimulate migration of cancer cells. FASEB J 35(7):e21692 34118087 10.1096/fj.202002558RR

[44] Patwardhan S, Mahadik P, Shetty O, Sen S (2021) ECM stiffness-tuned exosomes drive breast cancer motility through thrombospondin-1. Biomaterials 279:121185 34808560 10.1016/j.biomaterials.2021.121185

[45] Ndishabandi D, Duquette C, Billah GE, Reyes M, Duquette M, Lawler J, Kazerounian S (2014) Thrombospondin-1 modulates actin filament remodeling and cell motility in mouse mammary tumor cells in vitro. Discoveries (Craiova). 10.15190/d.2014.23 26273699 10.15190/d.2014.23 PMC4532438

[46] Sun S, Dong H, Yan T, Li J, Liu B, Shao P, Li J, Liang C (2020) Role of TSP-1 as prognostic marker in various cancers: a systematic review and meta-analysis. BMC Med Genet 21(1):139 32600280 10.1186/s12881-020-01073-3 PMC7325168

[47] Wang W, Wang X, Yao F, Huang C (2022) Lysyl oxidase family proteins: prospective therapeutic targets in cancer. Int J Mol Sci. 10.3390/ijms232012270 36293126 10.3390/ijms232012270 PMC9602794

[48] Cox TR, Erler JT (2013) Lysyl oxidase in colorectal cancer. Am J Physiol Gastrointest Liver Physiol 305(10):G659–G666 24008360 10.1152/ajpgi.00425.2012

[49] Liu Y, Zhang Y, Tan Z, Wang J, Hu Y, Sun J, Bao M, Huang P, Ge M, Chai YJ, Zheng C (2022) Lysyl oxidase promotes anaplastic thyroid carcinoma cell proliferation and metastasis mediated via BMP-1. Gland Surg 11(1):245–257 35242686 10.21037/gs-21-908 PMC8825512

[50] Wu X, Liu T, Fang O, Leach LJ, Hu X, Luo Z (2014) miR-194 suppresses metastasis of non-small cell lung cancer through regulating expression of BMP-1 and p27(kip1). Oncogene 33(12):1506–1514 23584484 10.1038/onc.2013.108

[51] Gonzalez-Angulo AM, Morales-Vasquez F, Hortobagyi GN (2007) Overview of resistance to systemic therapy in patients with breast cancer. Adv Exp Med Biol 608:1–22 17993229 10.1007/978-0-387-74039-3_1

[52] Tovar-Vidales T, Fitzgerald AM, Clark AF, Wordinger RJ (2013) Transforming growth factor-beta2 induces expression of biologically active bone morphogenetic protein-1 in human trabecular meshwork cells. Invest Ophthalmol Vis Sci 54(7):4741–4748 23788373 10.1167/iovs.13-12203 PMC3719445

[53] Yi M, Wu Y, Niu M, Zhu S, Zhang J, Yan Y, Zhou P, Dai Z, Wu K (2022) Anti-TGF-beta/PD-L1 bispecific antibody promotes T cell infiltration and exhibits enhanced antitumor activity in triple-negative breast cancer. J Immunother Cancer. 10.1136/jitc-2022-005543 36460337 10.1136/jitc-2022-005543 PMC9723957

[54] Kim BG, Malek E, Choi SH, Ignatz-Hoover JJ, Driscoll JJ (2021) Novel therapies emerging in oncology to target the TGF-beta pathway. J Hematol Oncol 14(1):55 33823905 10.1186/s13045-021-01053-x PMC8022551

[55] Bai X, Yi M, Jiao Y, Chu Q, Wu K (2019) Blocking TGF-beta signaling to enhance the efficacy of immune checkpoint inhibitor. Onco Targets Ther 12:9527–9538 31807028 10.2147/OTT.S224013 PMC6857659

[56] Batlle E, Massague J (2019) Transforming growth factor-beta signaling in immunity and cancer. Immunity 50(4):924–940 30995507 10.1016/j.immuni.2019.03.024 PMC7507121

[57] Mariathasan S, Turley SJ, Nickles D, Castiglioni A, Yuen K, Wang Y, Kadel EE III, Koeppen H, Astarita JL, Cubas R et al (2018) TGFbeta attenuates tumour response to PD-L1 blockade by contributing to exclusion of T cells. Nature 554(7693):544–548 29443960 10.1038/nature25501 PMC6028240

[58] Joshi A, Cao D (2010) TGF-beta signaling, tumor microenvironment and tumor progression: the butterfly effect. Front Biosci (Landmark Ed) 15(1):180–194 20036814 10.2741/3614

[59] Khoshakhlagh M, Soleimani A, Binabaj MM, Avan A, Ferns GA, Khazaei M, Hassanian SM (2019) Therapeutic potential of pharmacological TGF-beta signaling pathway inhibitors in the pathogenesis of breast cancer. Biochem Pharmacol 164:17–22 30905655 10.1016/j.bcp.2019.03.031

[60] Ahmadi A, Najafi M, Farhood B, Mortezaee K (2019) Transforming growth factor-beta signaling: tumorigenesis and targeting for cancer therapy. J Cell Physiol 234(8):12173–12187 30537043 10.1002/jcp.27955 PMC13484046

[61] Derynck R, Zhang YE (2003) Smad-dependent and Smad-independent pathways in TGF-beta family signalling. Nature 425(6958):577–584 14534577 10.1038/nature02006

[62] Zhang M, Wu J, Mao K, Deng H, Yang Y, Zhou E, Liu J (2017) Role of transforming growth factor-beta1 in triple negative breast cancer patients. Int J Surg 45:72–76 28754615 10.1016/j.ijsu.2017.07.080

[63] Shukla N, Naik A, Moryani K, Soni M, Shah J, Dave H (2022) TGF-beta at the crossroads of multiple prognosis in breast cancer, and beyond. Life Sci 310:121011 36179816 10.1016/j.lfs.2022.121011

[64] Teixeira AF, Ten Dijke P, Zhu HJ (2020) On-target anti-TGF-beta therapies are not succeeding in clinical cancer treatments: what are remaining challenges? Front Cell Dev Biol 8:605 32733895 10.3389/fcell.2020.00605 PMC7360684

[65] Colak S, Ten Dijke P (2017) Targeting TGF-beta signaling in cancer. Trends Cancer 3(1):56–71 28718426 10.1016/j.trecan.2016.11.008

