# Supplementary material for: Targeting BMP-1 enhances anti-tumoral effects of doxorubicin in metastatic mammary cancer: common and distinct features of TGF-β inhibition
Source: Breast Cancer Res Treat. 2025 Jan 10;210(3):563–74. doi: 10.1007/s10549-024-07592-4 (PMC11953206; doi:10.1007/s10549-024-07592-4)
Supplement: Supplementary file 1 — Supplementary file1 (PDF 726 KB) [file 10549_2024_7592_MOESM1_ESM.pdf]

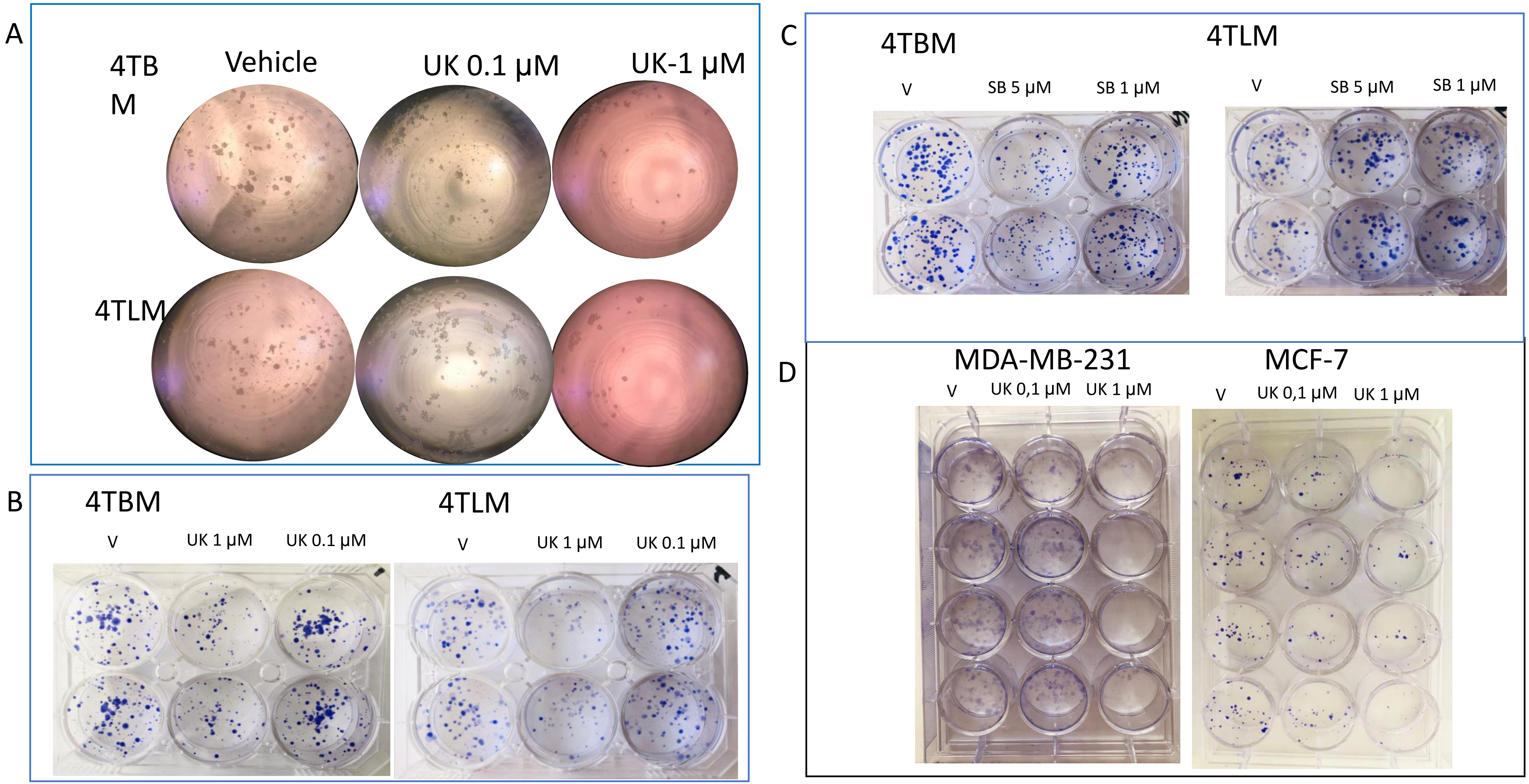

Supp Figure . Panel A demonstrates appearance of spheroids formed by 4TBM and 4TLM cells that are either untreated (Vehicle) or treated with BMP-1 inhibitor UK383367 (UK). Panel B shows the colonies of 4TBM and 4TLM cells following treatment with BMP-1 inhibitor. Panel C shows appearance of colonies of 4TBM and 4TLM cells that are either untreated (Vehicle) or treated with TGF-β inhibitor SB431542 (SB). Panel D demonstrates colony formation of MDA-MB-231 and MCF-7 cell that are either untreated (Vehicle) or treated with BMP-1 inhibitor UK383367 (UK).
